# Pulmonary function report capture and rule-based text indexing in an inpatient EHR cohort with recorded COPD

**DOI:** 10.3389/fmed.2026.1924553

**Published:** 2026-10-27

**Authors:** Kai Yang, Congcong Li, Wenjing Chen, Jiemin He, Maimaititusun Yalikun, Jingcheng Dong

**Affiliations:** 1 Department of Integrative Medicine, Huashan Hospital, Fudan University, Shanghai, China; 2 Institutes of Integrative Medicine, Fudan University, Shanghai, China

**Keywords:** COPD, data provenance, electronic health records, natural language processing, pulmonary function test, report capture, rule-based text indexing

## Abstract

**Background:**

Inpatient electronic health record (EHR) extracts may not contain pulmonary function reports stored in outpatient or laboratory systems. Before report text is used to refine a chronic obstructive pulmonary disease (COPD) research cohort, investigators need to know how much of that text is captured and whether this capture is selective. Herein, we audited pulmonary function report capture and built a transparent index of literal report terms.

**Methods:**

We conducted a single-center retrospective EHR study at Huashan Hospital, Fudan University, during a 3-year period between 2022 and 2025. The export contained 19,612 hospitalizations from 5,961 patients. EHR-recorded COPD was defined by prespecified diagnosis terms. Report capture meant that a pulmonary function report row was present in this inpatient analytic export; it did not indicate whether testing had ever been performed. Direct phrase rules indexed literal ItemResult text. The export did not contain International Classification of Diseases (ICD) codes, timestamps, structured pulmonary function values, historical local laboratory rules, reader metadata, or a clinician-annotated reference standard.

**Results:**

The main definition identified 6,398 hospitalizations with COPD wording from 2,981 patients. A pulmonary function report row was captured for 322 patients (10.8%), whereas 2,659 (89.2%) had no captured report row. For 278 patients, a report was linked to the same hospitalization record as COPD wording. Capture differed across sex, age, smoking documentation, respiratory diagnoses, and lung cancer strata. The 561 report rows in the full export were reduced to 216 unique normalized strings, a 61.5% reduction in template-level review units. In the same-hospitalization subset, common literal terms included ventilatory impairment (55.0%), mixed ventilatory impairment (50.0%), reduced respiratory muscle strength (48.2%), small-airway abnormality (44.6%), and explicit obstructive wording (31.7%). These are report-text frequencies and not physiologic prevalence estimates.

**Conclusion:**

Report rows were captured for only a small, selected subset, and therefore, this inpatient export cannot support full-cohort physiologic refinement without additional data linkage. The rule index organizes captured text for audit and review but is not a clinically validated classifier.

## Introduction

Chronic obstructive pulmonary disease (COPD) cohorts built from routinely collected electronic health record (EHR) data are often defined with diagnosis codes, diagnosis names, or both. These approaches are practical for large studies, but they do not establish postbronchodilator airflow obstruction. Global Initiative for Chronic Obstructive Lung Disease (GOLD) recommends spirometric confirmation, and European Respiratory Society/American Thoracic Society (ERS/ATS) standards describe how spirometry and other lung-function tests should be measured and interpreted (1–3). Reviews and validated algorithms also show that COPD cohort definitions vary across data sources and may not be directly comparable (4–6).

An additional problem is data provenance. A research extract from an inpatient system may contain diagnosis wording but omit pulmonary function values or reports held in an outpatient module, a pulmonary function laboratory system, or another data warehouse. In that setting, the absence of a report row does not show that testing was never performed. It shows only that the report was not captured in the extract being analyzed. This distinction is important because using the captured reports to refine a COPD cohort can remove most patients and create a selected subgroup.

Published estimates of documented or completed spirometry vary across settings and definitions. In a US EHR intervention study of patients with a COPD diagnosis and no prior spirometry, completion rates increased from 13.2% to 19.3% (7). In a 2025 primary-care registry study, 54% of 2,610 patients with recorded COPD had spirometry data (8). These figures are not directly comparable with each other or with an inpatient report-capture measure, but they show that the usable proportion depends strongly on the data source, time window, linkage, and denominator.

Natural language processing (NLP) has been used to identify clinical phenotypes in EHRs (9–11). Reviews also describe the use of clinical text extraction for cohort identification and research applications (12, 13). Prior pulmonary function studies have focused mainly on locating reports or extracting numeric measurements (14, 15). Automated interpretation of complete pulmonary function tests (PFTs) has also been studied (16), but the present export did not contain the underlying measurements. When an extract contains only short conclusion sentences, direct phrase indexing can organize the text for review. Its immediate value is operational: it can reduce the number of templates that must be inspected, identify records for clinician annotation, and document exactly which phrases were found. It is not a substitute for physiologic interpretation.

We therefore treated this work as a data-provenance and cohort-design audit. The objectives were to quantify pulmonary function report capture in an inpatient EHR cohort with recorded COPD, examine whether capture was selective across patient strata, build an auditable index of literal report terms, and explain how different capture levels should affect the design and interpretation of future COPD research.

## Materials and methods

### Study design and data source

This was a retrospective, single-center study of routinely collected inpatient EHR data from Huashan Hospital, Fudan University. The analytic export covered a 3-year period between 2022 and 2025 and contained one worksheet named Data with 19,612 hospitalization records from 5,961 patients and 70 columns. Patient and hospitalization identifiers were used only to link records and were not included in any output.

The study was approved by the Ethics Committee of the School of Life Sciences, Fudan University (approval number SLH2026212-001). The committee waived the requirement for informed consent because the study used retrospective, de-identified EHR data. Reporting followed Strengthening the Reporting of Observational Studies in Epidemiology (STROBE) and REporting of studies Conducted using Observational Routinely collected health Data (RECORD) guidelines (17, 18).

### Meaning of report capture and analysis units

Pulmonary function report capture was defined as the presence of at least one report row identified by the report-name or project-name fields in this inpatient analytic export. Capture is a property of the exported dataset. It is not a measure of whether a patient ever underwent pulmonary function testing, whether a test was completed during an exacerbation admission, or whether clinical care met a quality standard. A patient without a captured report could have undergone testing in an outpatient clinic, in another hospital, or in a laboratory system that was not linked to this export.

Patient-level clinical characteristics and any-report capture were summarized across the export. The file did not contain encounter dates or report timestamps, and therefore, we could not determine whether a report preceded or followed the first COPD documentation or whether testing occurred during a stable period or an acute admission. The primary text-index analysis used patients whose COPD wording and pulmonary function report shared the same hospitalization identifier. This link shows co-occurrence within a hospitalization record but not the order or exact timing of testing. If a hospitalization contained more than one report row, non-duplicate ItemResult text was combined at the hospitalization level; patient-level labels were then aggregated across eligible hospitalizations.

### Definition of EHR-recorded COPD

The main definition searched three diagnosis text fields: indexed diagnosis, outpatient or emergency diagnosis, and preliminary diagnosis. A hospitalization was classified as containing COPD wording when one of these fields contained an English abbreviation [COPD or chronic obstructive lung disease (COLD)] or a prespecified local Chinese term for chronic obstructive pulmonary disease, chronic obstructive lung disease, or chronic bronchitis with emphysema. The exact terms are listed in Data Sheet 1, and a patient entered the cohort after at least one qualifying hospitalization. Because the definition depended on recorded wording, it could omit differently worded records and did not establish spirometry-confirmed COPD.

The export did not contain a separate International Classification of Diseases (ICD) diagnosis-code field, and no J41-J44 strings were found in the three diagnosis fields. We therefore could not construct or directly compare an ICD-based cohort. Sensitivity definitions included a strict definition limited to the indexed diagnosis field, a broad definition that also searched chief complaint, present illness, and past history, and an expanded term definition that added local wording for chronic obstructive bronchitis and chronic bronchitis with emphysema.

To describe the cohort, COPD-coded hospitalizations were also searched for wording related to acute exacerbation, respiratory infection or pneumonia, and respiratory failure. These groups were not mutually exclusive and did not establish that COPD was the primary reason for admission.

### Identification and indexing of pulmonary function report text

Pulmonary function reports were identified when either the report name or project name equaled the local term for pulmonary function. This identified 561 report rows in the full export. ItemResult was the only populated report conclusion field. The examination-findings and ItemShow1 fields were empty. Structured pulmonary function values were not available.

ItemResult text was normalized before rule matching. Carriage returns were converted to line breaks, repeated spaces were removed within each line, blank lines were dropped, and original line boundaries were retained. Two local wording variants were normalized, and each line was divided into short clauses at common Chinese and English punctuation marks.

Rules indexed explicit wording for ventilatory impairment, mixed ventilatory impairment, obstruction-predominant wording, restrictive wording, small-airway abnormality, diffusing impairment, reduced inspiratory or expiratory muscle strength, increased respiratory central drive, increased total airway resistance, and positive or negative bronchodilator-test wording. Normal statements were retained only when an abnormal term was absent from the same segment. Explicit obstructive wording required the literal obstruction term; increased total airway resistance was counted separately. Poor-cooperation or reference-only wording was flagged. Table 1 summarizes report capture and rule-based text indexing. The full dictionary and exclusions are provided in Data Sheet 1.

**Table 1:** Summary of report capture and rule-based text indexing.

Domain or step	Rule logic	Safeguard or interpretation
Report capture	Report name or project name equaled the local pulmonary-function term	Measures the presence of a report row in this inpatient export, not lifetime testing or care quality
Text normalization	Preserve line breaks; remove repeated spaces and blank lines; normalize two local variants	Exact source terms are in Data Sheet 1
Ventilatory impairment	Direct match to ventilatory-impairment wording	No calculation from numeric values
Normal ventilation	Normal wording in a segment without impairment wording	Segment-level exclusion
Explicit obstructive wording	Literal obstruction term in the report text	Total airway resistance is indexed separately
Mixed ventilatory impairment	Mixed wording and ventilatory-impairment wording in the same segment	Report term only; not recalculated
Explicit restrictive wording	Restrictive wording linked to ventilation in the same segment	Small-airway wording is excluded
Small-airway abnormality	Small-airway wording plus air trapping, abnormality, reduction, or impairment in the same segment	Normal wording is stored separately
Diffusing impairment	Diffusing-function wording plus reduced or decreased wording in the same segment	Not-tested wording is stored separately
Respiratory muscle strength	Direct match to reduced inspiratory- or expiratory-muscle-strength wording	Patient positive if either term is present
Respiratory central drive	Direct match to increased respiratory-central-drive wording	Local wording normalized first
Total airway resistance	Direct match to increased total-airway-resistance wording	Not included in explicit obstruction
Bronchodilator test	Positive or negative wording near bronchodilator-test wording	No inference when the test is not mentioned
Quality flag	Poor-cooperation or reference-only wording	Used for sensitivity analysis only
Patient aggregation	Term present if found in any report linked to a COPD-coded hospitalization for that patient	Primary text-index analysis *N* = 278

The full phrase dictionary, including the original Chinese source terms and normalization rules, is provided in Data Sheet 1. The index retrieves literal wording and is not a physiologic classifier.

The rules did not calculate physiologic categories from numeric thresholds. A negative rule result means that the prespecified literal term was not found; it does not mean that the physiologic abnormality was absent. No clinician-annotated reference standard was available, and hence, accuracy, sensitivity, specificity, precision, recall, F1 score, and area under the curve were not calculated.

### Published interpretation frameworks and limits of local reconstruction

Published international standards were reviewed to identify the measurements normally required for pulmonary function interpretation (2, 3). The Chinese pulmonary function guideline and the ERS respiratory muscle statement were also used for context (19, 20). Obstruction and mixed impairment require spirometric ratios and, for mixed or restrictive patterns, lung-volume information; diffusing impairment requires diffusing capacity of the lung for carbon monoxide (DLCO) with an appropriate reference equation; respiratory muscle strength requires measured inspiratory or expiratory pressures; and severity depends on the chosen reference system. The Chinese 2018 primary-care guideline describes a small-airway rule based on at least two of forced expiratory flow at 50% of FVC (FEF50), FEF75, and FEF25-75 being below 65% predicted when the main spirometric indices are normal (19).

These published frameworks were not treated as the exact Huashan Hospital rules. The analytic export and available study files did not contain the historical pulmonary function laboratory standard operating procedure (SOP) version used during 2022–2025, the reference equations, instrument or software settings, numeric measurements, or reader metadata. We therefore could not reconstruct or verify the local thresholds used to assign each report conclusion. Table 2 separates published physiologic frameworks from what was available in this study. Severity words remain documented in the rule dictionary, but severity frequencies were removed from the main results because their local numeric basis could not be verified.

**Table 2:** Published pulmonary function interpretation frameworks and data available in the current export.

Report term or domain	Published physiologic framework	What was available in this export	Treatment in this study
Obstructive ventilatory impairment	Reduced FEV1/FVC relative to the chosen diagnostic or LLN framework; COPD confirmation requires postbronchodilator spirometry (1–3)	Final conclusion text only; no FEV1/FVC, bronchodilator values, reference equation, or local threshold	Literal obstruction wording only; not GOLD confirmation
Restrictive or mixed impairment	Restriction requires lung-volume evidence such as reduced TLC; mixed impairment requires obstructive and restrictive evidence (2, 19)	No TLC, FVC, FEV1/FVC, or local calculation rules	Literal restrictive or mixed wording only
Small-airway abnormality	The Chinese 2018 guideline describes at least two of FEF50, FEF75, and FEF25-75 below 65% predicted when main spirometric indices are normal (19)	No flow measurements, predicted values, reference equation, or local SOP	Literal small-airway wording only
Diffusing impairment	DLCO should be interpreted against an appropriate reference distribution or LLN (2)	No DLCO, hemoglobin correction details, reference equation, or local threshold	Literal diffusing-impairment wording only
Respiratory muscle weakness	Requires measured inspiratory and/or expiratory pressures with appropriate reference limits (20)	No MIP/MEP values, reference limits, or reader metadata	Literal reduced-muscle-strength wording only
Increased respiratory central drive	Requires a measured drive variable and a stated local reference or interpretive method	No drive measurement, instrument method, reference interval, or local SOP	Literal increased-central-drive wording only
Severity	Modern ERS/ATS interpretation uses z-score-based severity; Chinese and local schemes may use percent-predicted categories (2, 19)	No numeric values or verified historical local severity scheme	Severity frequencies removed from the main results

FEF50, forced expiratory flow at 50% of FVC; FEF75, forced expiratory flow at 75% of FVC; FEF25-75, mean forced expiratory flow between 25% and 75% of FVC; FEV1, forced expiratory volume in 1 s; FVC, forced vital capacity; LLN, lower limit of normal; MEP, maximal expiratory pressure; MIP, maximal inspiratory pressure; TLC, total lung capacity.

Published frameworks are provided to show what numeric data would normally be required. They are not presented as the verified historical Huashan Hospital SOP.

### Clinical variables and data-quality decisions

Clinical variables were summarized at the patient level. Sex was assigned from the most frequently recorded value for each patient. Age and body mass index (BMI) were summarized from physiologically plausible values. Comorbidities and hospitalization features were identified from recorded diagnosis wording and were used only to examine whether report capture was selective.

Smoking documentation was classified as documented smoking history, smoking denied in EHR text, or unclear/not documented. Current and former smoking were not separated because the available templates did not support that distinction reliably. Alcohol status and sparse blood pressure and hemoglobin values were not used in the revised comparisons. An exploratory physiologic-range check suggested that the exported heart-rate and respiratory-rate columns may have been interchanged, but this could not be verified against the source system. We therefore did not relabel, correct, summarize, compare, or otherwise use either variable. Any future use requires source-system verification.

### Statistical analysis

Continuous variables are reported as median and interquartile range, with the number of available observations. Categorical variables are reported as count and percentage. Captured and uncaptured groups were compared with the Mann–Whitney U test for continuous variables and the chi-square test or Fisher’s exact test for categorical variables, as appropriate. These *P*-values are descriptive and unadjusted.

Wilson 95% confidence intervals (CIs) were calculated for capture proportions and literal report-term proportions. Sex-stratified comparisons were exploratory; two-sided Fisher’s exact tests were used because only 24 women were included in the same-hospitalization analysis. Sensitivity analyses used same-hospitalization report episodes, any-admission patient-level data, all report rows, a quality-filtered set, an acute-exacerbation subset, and alternative COPD text definitions. Analyses were performed with Python 3.13 and SciPy 1.17.0.

## Results

### Cohort and report-capture audit

The export included 19,612 hospitalization records from 5,961 patients. The main diagnosis-text definition identified 6,398 hospitalizations containing COPD wording from 2,981 patients. Among the COPD-coded hospitalizations, 2,604 (40.7%) contained acute-exacerbation wording, 1,151 (18.0%) contained respiratory infection or pneumonia wording, and 467 (7.3%) contained respiratory-failure wording. The groups overlap, and 3,065 hospitalizations (47.9%) contained none of these three wording groups.

A pulmonary function report row was captured for 322 of the 2,981 patients (10.8%). Conversely, 2,659 patients (89.2%) had no pulmonary function report row in this inpatient export. The 322 captured patients contributed 430 report rows. For 278 patients, COPD wording and a report were linked to the same hospitalization identifier (9.3% of the full cohort and 86.3% of captured patients), contributing 335 report episodes. The remaining 44 captured patients had report text only in a different hospitalization record and were excluded from the primary text-index analysis (Figure 1).

**Figure 1:**
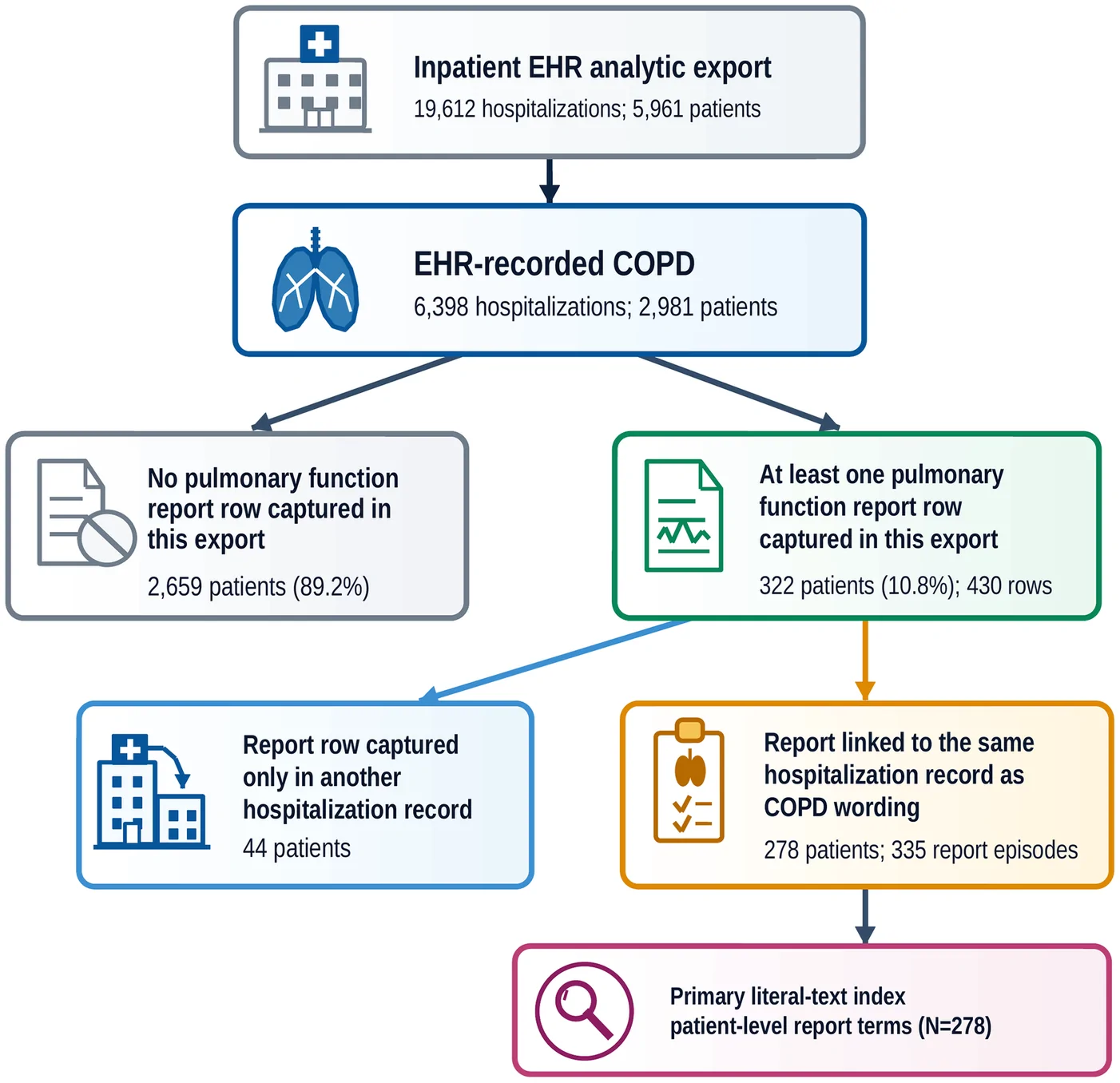
Cohort flow and pulmonary function report capture. Among 2,981 patients with EHR-recorded COPD, 322 (10.8%) had at least one report row in the inpatient export; 278 had COPD wording and a report linked to the same hospitalization identifier and formed the primary text-index subset. Report capture indicates presence in this export, not lifetime testing or test timing.

### Selection in report capture

The report capture rate was 11.5% in men and 7.2% in women. It was 11.6% in patients younger than 65 years, 11.8% in those aged 65–79 years, and 8.0% in those aged 80 years or older. The capture rate was also higher among patients with documented smoking history or lung cancer and lower among patients with respiratory-failure wording. These differences are descriptive and may reflect care setting, referral, data linkage, or documentation practice rather than testing need or quality (Figure 2).

**Figure 2:**
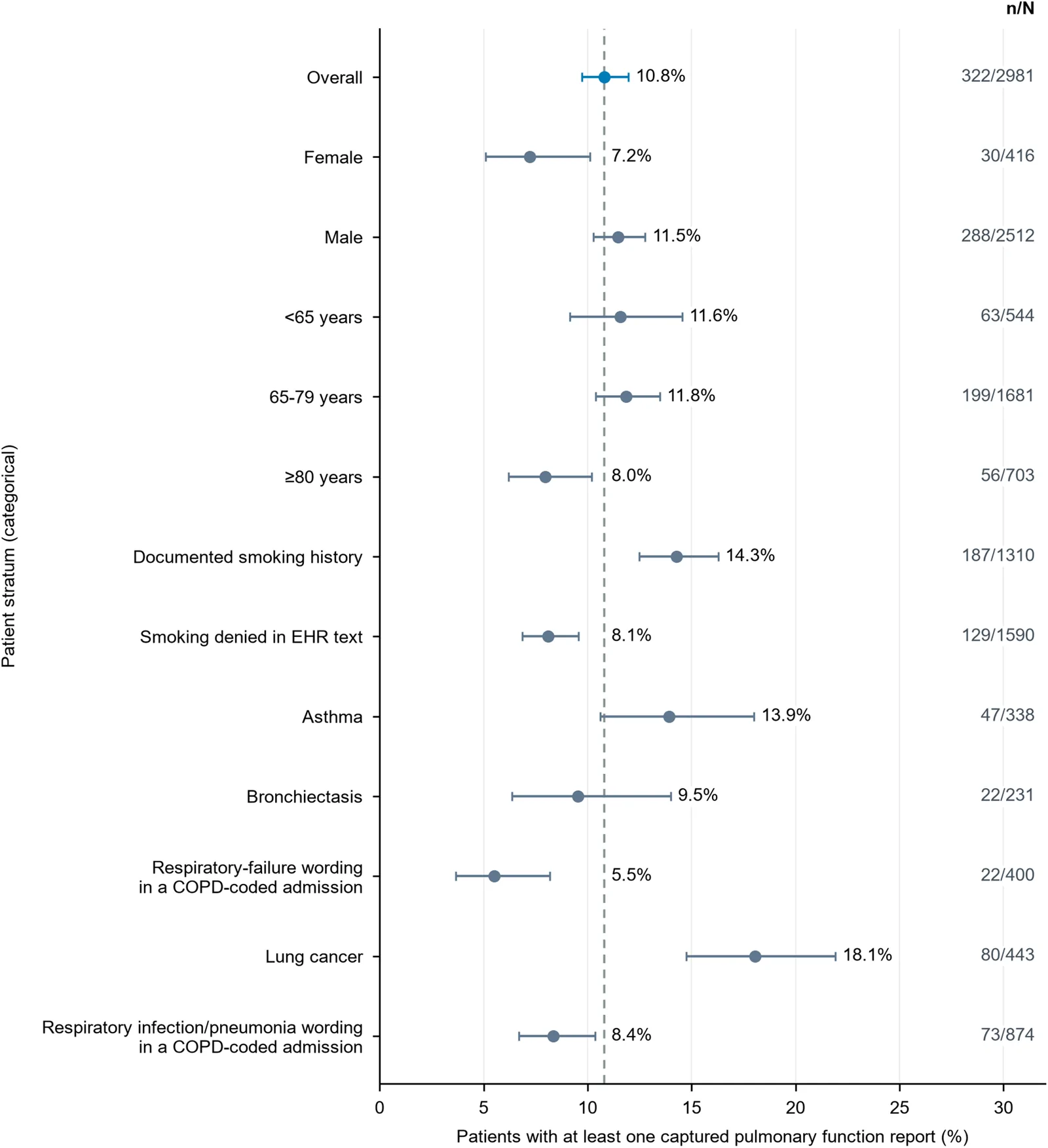
Pulmonary function report capture by selected EHR strata. The points show the percentage of patients with at least one captured report row, and the horizontal bars show Wilson 95% confidence intervals. The dashed line marks the overall capture proportion of 10.8%, and the values on the right are n/N. Comparisons are descriptive and unadjusted.

The median age of the cohort was 72 years, and 84.3% of patients were men. Captured patients had more total hospitalizations and more COPD-coded hospitalizations than uncaptured patients. Recorded respiratory infection or pneumonia and respiratory failure were less common in the captured group, whereas lung cancer and interstitial lung disease were more common. These unadjusted comparisons show that the captured subset was not a simple random sample of the full diagnosis-text cohort (Table 3).

**Table 3:** Patient-level characteristics by pulmonary function report capture in the inpatient analytic export.

Characteristic	Overall (*N* = 2,981)	Captured report (*n* = 322)	No captured report (*n* = 2,659)	*P*-value
Age, years	72.0 (66.0–79.0); *n* = 2,928	71.0 (66.0–77.0); *n* = 318	72.0 (66.5–79.0); *n* = 2,610	0.057
Sex				0.026
Male	2,512 (84.3)	288 (89.4)	2,224 (83.6)	
Female	416 (14.0)	30 (9.3)	386 (14.5)	
Unknown	53 (1.8)	4 (1.2)	49 (1.8)	
Number of hospitalizations	1 (1–2)	2 (1–5)	1 (1–2)	<0.001
Number of COPD-coded hospitalizations	1 (1–1)	1 (1–2)	1 (1–1)	<0.001
Body mass index, kg/m^2^	22.5 (19.9–25.1); *n* = 2,495	22.7 (20.3–25.0); *n* = 296	22.5 (19.8–25.2); *n* = 2,199	0.242
Smoking documentation				<0.001
Documented smoking history	1,310 (43.9)	187 (58.1)	1,123 (42.2)	
Smoking denied in EHR text	1,590 (53.3)	129 (40.1)	1,461 (54.9)	
Unclear/not documented	81 (2.7)	6 (1.9)	75 (2.8)	
Acute-exacerbation wording in any COPD-coded admission	1,218 (40.9)	120 (37.3)	1,098 (41.3)	0.165
Respiratory infection/pneumonia wording in any COPD-coded admission	874 (29.3)	73 (22.7)	801 (30.1)	0.006
Respiratory-failure wording in any COPD-coded admission	400 (13.4)	22 (6.8)	378 (14.2)	<0.001
Hypertension	1,354 (45.4)	150 (46.6)	1,204 (45.3)	0.657
Diabetes	575 (19.3)	60 (18.6)	515 (19.4)	0.752
Coronary heart disease	585 (19.6)	68 (21.1)	517 (19.4)	0.475
Heart failure	377 (12.6)	37 (11.5)	340 (12.8)	0.509
Atrial fibrillation	271 (9.1)	26 (8.1)	245 (9.2)	0.502
Cerebrovascular disease	422 (14.2)	44 (13.7)	378 (14.2)	0.789
Bronchiectasis	231 (7.7)	22 (6.8)	209 (7.9)	0.515
Asthma	338 (11.3)	47 (14.6)	291 (10.9)	0.051
Interstitial lung disease	73 (2.4)	15 (4.7)	58 (2.2)	0.007
Lung cancer	443 (14.9)	80 (24.8)	363 (13.7)	<0.001

Values are median (interquartile range), with *n* shown when data are missing, or *n* (%). *P*-values are descriptive and unadjusted. Capture means at least one pulmonary function report row in this inpatient export. Diagnosis and smoking variables describe EHR documentation and were not independently validated.

### Literal report-text index

The 561 report rows in the full export contained 216 unique normalized ItemResult strings. Reviewing unique strings rather than every row would reduce template-level review units by 345, or 61.5%. Every unique string produced at least one prespecified abnormal term, normal statement, or quality flag. This is a dictionary-reach audit and not an accuracy estimate. Figure 3 summarizes how report-capture auditing informs cohort design, text indexing, and validation requirements.

**Figure 3:**
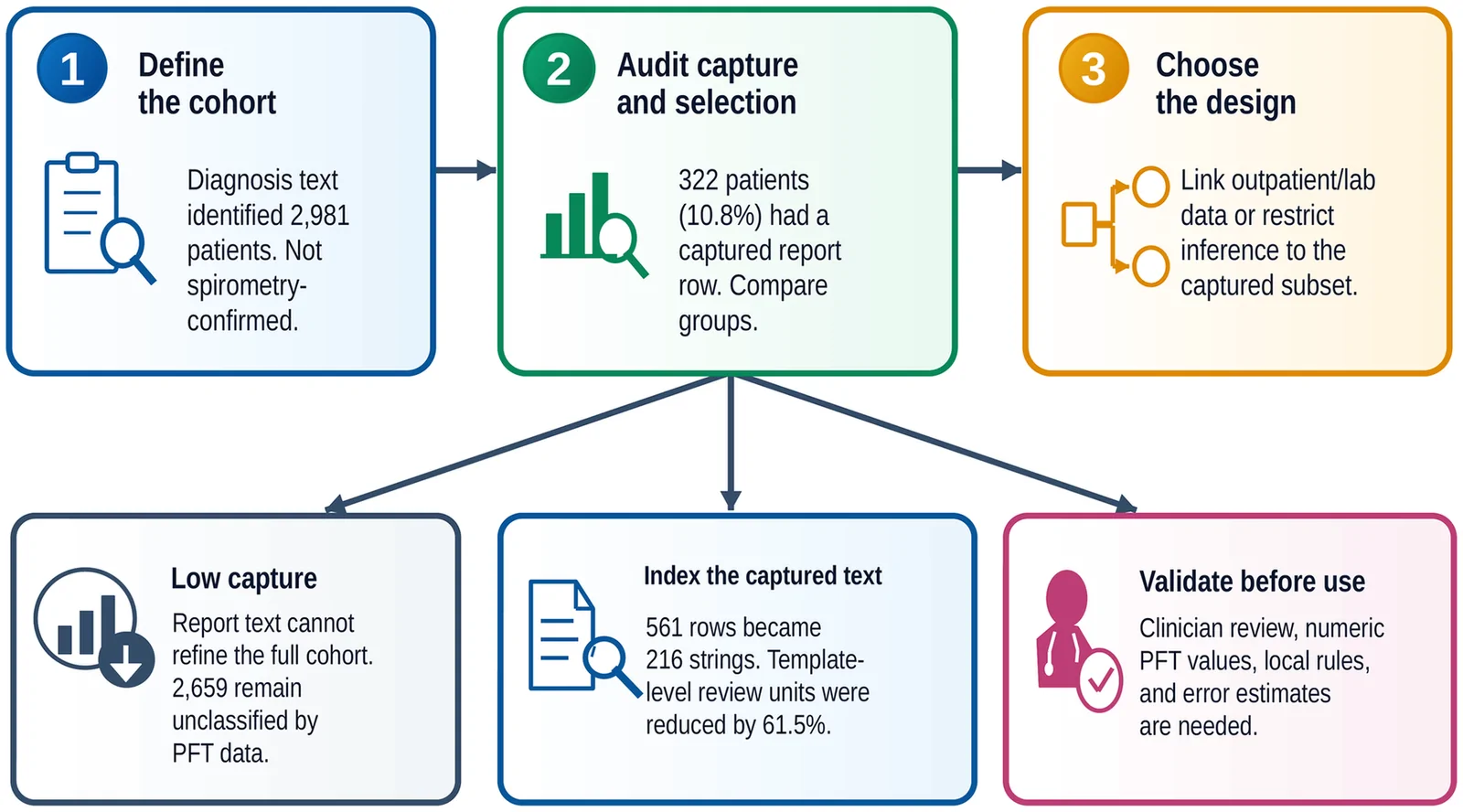
How report capture informs EHR cohort design. The workflow shows how a diagnosis-text cohort is audited for report capture and selection, how captured text can be indexed, and why additional data linkage and clinical validation remain necessary.

The primary same-hospitalization analysis included 278 patients. Literal ventilatory-impairment wording was present in 153 patients (55.0%, 95% CI 49.2–60.8), mixed ventilatory-impairment wording in 139 (50.0%, 44.2–55.8), reduced inspiratory and/or expiratory muscle-strength wording in 134 (48.2%, 42.4–54.1), increased respiratory-central-drive wording in 129 (46.4%, 40.6–52.3), and small-airway-abnormality wording in 124 (44.6%, 38.9–50.5). Explicit obstructive wording was present in 88 patients (31.7%, 26.5–37.3), diffusing-impairment wording in 85 (30.6%, 25.5–36.2), and obstruction-predominant wording in 78 (28.1%, 23.1–33.6). These percentages describe the literal text in captured reports and must not be interpreted as the prevalence of physiologic abnormalities (Table 4 and Figure 4).

**Table 4:** Literal pulmonary function report terms in the same-hospitalization subset, overall, and by sex.

Literal report term	Overall (*N* = 278)	Men (*n* = 250)	Women (*n* = 24)	Fisher’s exact *P*
Ventilatory impairment	153/278 (55.0) [49.2–60.8]	138/250 (55.2) [49.0–61.2]	13/24 (54.2) [35.1–72.1]	1.000
Mixed ventilatory impairment	139/278 (50.0) [44.2–55.8]	126/250 (50.4) [44.2–56.5]	11/24 (45.8) [27.9–64.9]	0.831
Explicit obstructive wording	88/278 (31.7) [26.5–37.3]	81/250 (32.4) [26.9–38.4]	5/24 (20.8) [9.2–40.5]	0.357
Obstruction-predominant wording	78/278 (28.1) [23.1–33.6]	72/250 (28.8) [23.5–34.7]	4/24 (16.7) [6.7–35.9]	0.241
Explicit restrictive wording	11/278 (4.0) [2.2–6.9]	8/250 (3.2) [1.6–6.2]	3/24 (12.5) [4.3–31.0]	0.061
Small-airway abnormality	124/278 (44.6) [38.9–50.5]	111/250 (44.4) [38.4–50.6]	11/24 (45.8) [27.9–64.9]	1.000
Diffusing impairment	85/278 (30.6) [25.5–36.2]	80/250 (32.0) [26.5–38.0]	4/24 (16.7) [6.7–35.9]	0.164
Diffusing function not tested	36/278 (12.9) [9.5–17.4]	32/250 (12.8) [9.2–17.5]	3/24 (12.5) [4.3–31.0]	1.000
Reduced inspiratory and/or expiratory muscle strength	134/278 (48.2) [42.4–54.1]	121/250 (48.4) [42.3–54.6]	11/24 (45.8) [27.9–64.9]	0.834
Increased respiratory central drive	129/278 (46.4) [40.6–52.3]	116/250 (46.4) [40.3–52.6]	11/24 (45.8) [27.9–64.9]	1.000
Increased total airway resistance	18/278 (6.5) [4.1–10.0]	15/250 (6.0) [3.7–9.7]	3/24 (12.5) [4.3–31.0]	0.201
Poor-cooperation/reference-only wording	16/278 (5.8) [3.6–9.1]	13/250 (5.2) [3.1–8.7]	2/24 (8.3) [2.3–25.8]	0.629

Cells show n/N (%) [Wilson 95% CI]. Overall estimates include four patients with unknown sex. *P*-values are two-sided Fisher’s exact tests comparing women and men and are descriptive. A term-negative report may contain the finding in another form; the table does not show physiologic prevalence or extraction accuracy.

**Figure 4:**
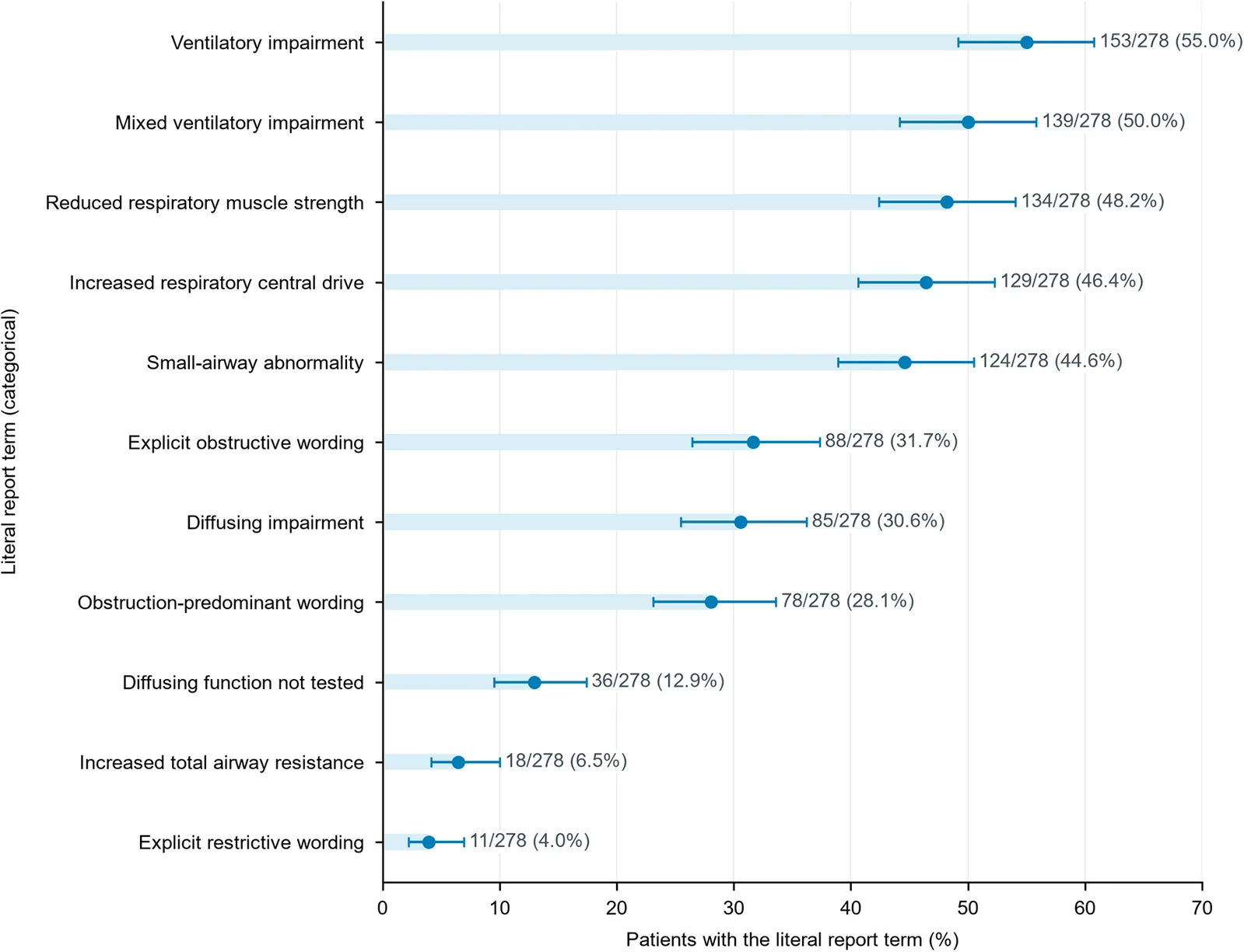
Literal pulmonary function report terms in the same-hospitalization subset. The points show the percentage of 278 patients with each prespecified literal term, and the horizontal bars show Wilson 95% confidence intervals. A term-negative report may contain the finding in another form. The figure describes captured text, not physiologic prevalence or clinical accuracy.

The same-hospitalization subset included 250 men, 24 women, and four patients with unknown sex. Because the female subgroup was small, the sex-stratified estimates had wide confidence intervals and were considered exploratory. These data do not support conclusions about sex differences in pulmonary physiology or report wording (Table 4).

### Sensitivity analyses

The main report-term pattern was similar when the unit was a same-hospitalization report episode (*N* = 335), when any captured report from an EHR-recorded COPD patient was used at the patient level (*N* = 322), and when all report rows were analyzed (*N* = 430). Alternative diagnosis-text definitions changed the same-hospitalization denominator from 274 to 286 patients but did not materially change the report-term frequencies.

In the acute-exacerbation same-hospitalization subset (*N* = 93), explicit obstructive wording was present in 24.7%, small-airway-abnormality wording in 40.9%, diffusing-impairment wording in 31.2%, reduced respiratory-muscle-strength wording in 55.9%, and increased respiratory-central-drive wording in 52.7%. The quality-filtered set was smaller, and percentage changes partly reflected its selected denominator (Table 5).

**Table 5:** Sensitivity analyses for key literal pulmonary function report terms.

Analysis	Unit	N	Ventilatory impairment	Mixed ventilatory impairment	Explicit obstructive wording	Small-airway abnormality	Diffusing impairment	Reduced muscle strength	Increased central drive
Same-hospitalization patient-level analysis (primary)	Patients	278	153 (55.0)	139 (50.0)	88 (31.7)	124 (44.6)	85 (30.6)	134 (48.2)	129 (46.4)
Same-hospitalization report episodes	Report episodes	335	167 (49.9)	150 (44.8)	94 (28.1)	135 (40.3)	99 (29.6)	155 (46.3)	145 (43.3)
Any-captured-report patient-level analysis	Patients	322	183 (56.8)	172 (53.4)	104 (32.3)	148 (46.0)	102 (31.7)	156 (48.4)	151 (46.9)
All report rows from EHR-recorded COPD patients	Report rows	430	226 (52.6)	203 (47.2)	120 (27.9)	181 (42.1)	127 (29.5)	190 (44.2)	178 (41.4)
Quality-filtered same-hospitalization patients	Patients	243	153 (63.0)	139 (57.2)	88 (36.2)	124 (51.0)	85 (35.0)	98 (40.3)	95 (39.1)
Acute-exacerbation same-hospitalization patients	Patients	93	43 (46.2)	40 (43.0)	23 (24.7)	38 (40.9)	29 (31.2)	52 (55.9)	49 (52.7)
Strict diagnosis-field definition	Patients	274	152 (55.5)	139 (50.7)	87 (31.8)	123 (44.9)	83 (30.3)	130 (47.4)	125 (45.6)
Broad diagnosis-plus-narrative definition	Patients	286	159 (55.6)	145 (50.7)	92 (32.2)	126 (44.1)	87 (30.4)	137 (47.9)	132 (46.2)
Expanded diagnosis-term definition	Patients	278	153 (55.0)	139 (50.0)	88 (31.7)	124 (44.6)	85 (30.6)	134 (48.2)	129 (46.4)

Values are *n* (%). The quality-filtered set excludes poor-cooperation/reference-only report episodes and episodes with diffusing function not tested. Alternative COPD definitions are based on recorded text and do not imply spirometry-confirmed COPD.

## Discussion

This study evaluated data provenance rather than pulmonary function test utilization. A report row was captured for 10.8% of patients with EHR-recorded COPD, while the remaining 89.2% cannot be classified as never tested because outpatient, external, or laboratory-system reports may be missing. The estimate therefore describes this analytic export and should not be used to judge test ordering, completion during exacerbations, or care quality.

The audit shows the consequence for study design. Requiring a captured report would exclude almost nine of every 10 patients and select a subgroup that differed from the uncaptured cohort. Researchers should therefore link outpatient or laboratory data, limit inference to the captured subset, or avoid report text as a full-cohort physiologic criterion. Data-quality teams can use the same workflow to locate linkage gaps and underrepresented groups.

We cannot determine whether the 10.8% capture rate is unique to Huashan Hospital. Published proportions vary widely, but their definitions are different. Wang et al. measured completed spirometry among patients with a COPD diagnosis and no prior test and reported 13.2% before and 19.3% after an EHR intervention (7). Vila et al. found spirometry data for 54% of patients with recorded COPD in a linked primary-care registry (8). Our denominator, setting, time window, and outcome were different: we measured report rows captured in an inpatient export. The comparison supports the general point that data architecture and linkage strongly affect the usable proportion; it does not rank hospitals.

No universal capture threshold makes report data suitable for cohort refinement. At 50% capture, the linked sample and statistical power would increase, but half of the cohort would still be missing and selection could remain. Researchers should report the denominator, compare captured and uncaptured patients, assess missingness, and validate content. Coverage and accuracy are separate properties.

The rule index addresses only the captured text. It maps literal phrases and reduces 561 report rows to 216 unique strings, creating an auditable sampling frame. It does not establish whether a report conclusion is clinically correct or whether a negative rule result means normal physiology. Therefore, Tables 4, 5 describe text frequency and not clinical prevalence or diagnostic performance.

The exact local pulmonary function criteria remain an important limitation. Published Chinese and ERS/ATS frameworks show which measurements and reference systems are normally needed, but they cannot substitute for the historical Huashan Hospital SOP. The export lacked the SOP version, reference equations, instrument settings, numeric values, and reader metadata. Therefore, we removed severity distributions and did not recalculate obstruction, restriction, diffusion loss, muscle weakness, or central drive from thresholds. If the historical rules become available, they should be documented and linked to numeric reports before clinical use.

The strengths of this study include a clear definition of report capture, separation of patient-, hospitalization-, and report-level units, a complete phrase dictionary, a same-hospitalization primary subset, and sensitivity analyses across cohort definitions. Limitations include the diagnosis-text cohort, absence of ICD codes and timestamps, the small female subgroup, lack of a clinician-annotated reference standard, missing numeric values and verified local laboratory rules, single-center design, and no outcome analysis. The exploratory heart-rate and respiratory-rate column check was not verified against the source system, and those variables were not used. These limitations restrict generalizability and preclude conclusions about timing, sex differences, diagnosis, prognosis, or care quality.

Formal validation should use a stratified sample of common and uncommon templates, quality-flagged reports, and reports from different periods or readers. Two independent pulmonary-function reviewers should label the target terms and resolve disagreements. Numeric spirometry, lung volumes, DLCO, respiratory pressures, the relevant drive measure, reference equations, and the local SOP should then be linked. Accuracy measures and clinical uses should be considered only after this work.

## Conclusion

In this inpatient export, pulmonary function report rows were captured for 10.8% of patients with EHR-recorded COPD. The audit quantifies denominator loss and selection and shows that full-cohort physiologic refinement requires additional outpatient or laboratory data linkage. The rule-based index is a reviewable map of captured text and not a clinically validated classifier.

## Data Availability

The data analyzed in this study are subject to the following licenses/restrictions: The datasets are subject to restrictions due to patient privacy protection and institutional data governance requirements of Huashan Hospital, Fudan University. Individual-level electronic medical record data cannot be made publicly available. Access may be granted upon reasonable request to the corresponding author and with explicit permission from the hospital’s data governance authority. Requests to access these datasets should be directed to Jingcheng Dong, jcdong2004@126.com. Aggregate results, the rule dictionary, and analysis tables are provided in Data Sheet 1. Data Sheet 1 contains the diagnosis and report-text rule dictionary, cohort-definition audits, the published-framework table, aggregate results, and source data for the figures. No individual-level EHR data are included.
